# Role of mesenchymal stem cell-derived exosomes in the regeneration of different tissues

**DOI:** 10.1186/s13036-024-00431-6

**Published:** 2024-06-06

**Authors:** Defa Huang, Haibin Shen, Fangfang Xie, Die Hu, Qing Jin, Yuexin Hu, Tianyu Zhong

**Affiliations:** 1 Laboratory Medicine, First Affiliated Hospital of Gannan Medical University, Ganzhou, China; 2 Precision Medicine Center, First Affiliated Hospital of Gannan Medical University, Ganzhou, China

**Keywords:** Mesenchymal stem cell, Exosomes, Tissue regeneration

## Abstract

Exosomes are nanovesicles with multiple components used in several applications. Mesenchymal stem cells (MSCs) are well known for their great potential in clinical applications. MSC-derived exosomes (MSC-Exos) have been shown to mediate tissue regeneration in various diseases, including neurological, autoimmune, and inflammatory diseases, cancer, ischemic heart disease, lung injury, and liver fibrosis. They can modulate the immune response by interacting with immune effector cells in the presence of anti-inflammatory compounds and are involved in intercellular communication through various types of cargo. This review summarizes the MSC-Exos-mediated tissue regeneration in various diseases, including neurological, cardiovascular, liver, kidney, articular cartilage, and oral tissue applications. In addition, we discuss the challenges and prospects of MSC-Exos in tissue regeneration.

## Introduction

Tissue regeneration refers to the replacement or repair of necrotic, damaged, or aged tissues with new tissue [1]. Regenerative medicine has faced numerous challenges in search for better alternatives to ensure effective treatments to accelerate the tissue regeneration process without altering its phases, especially for wound healing, in clinical, morphophysiological, and molecular environments [2–4]. Instead of whole-organ/tissue transplantation, cell therapy, a new approach, is used, where cells with immunomodulatory properties are used. Mesenchymal stem cells (MSCs) are an important resource for cell therapy and have been widely used for various applications, such as treating diabetic wounds and ulcers and regenerating several types of tissue [5–8]. MSCs can modulate immune cell activity and stimulate tissue regeneration, thus playing an increasingly important role in the treatment of many diseases [9–11]. The therapeutic effect is achieved in two main ways: direct mediation and induction of changes in the disease environment through the release of soluble factors [12]. When these soluble factors are released, they are encapsulated in a membrane structure similar to the cell membrane that form vesicles, which protects the stable presence and activity of these factors in the extracellular environment, and, the membrane structure can easily fuse with the cell membrane of other cells, facilitating the regulation of these inducible factors in other cells. These vesicle structures were classified according to their size into microvesicles (0.1–1 mm in diameter), exosomes (50–150 nm in diameter), and apoptotic vesicles (1–2 mm in diameter) [13].

Exosomes are produced and released extracellularly by almost all cell types and are widely distributed in various body fluids, such as urine, blood, breast milk, saliva, cerebrospinal fluid, amniotic fluid, and semen [14–17]. Exosomes contain several regulatory factors, such as cytokines, growth factors, signaling lipids, messenger RNAs (mRNA), and non-coding RNAs (miRNAs), which play important roles in regulating other cellular processes in the surrounding environment by MSCs [18–20]. The RNA or proteins in MSC derived exosomes (MSC-Exos) can play a cell-homing role and regulate cell proliferation and differentiation, which can limit damage, regulate immune responses, and promote self-repair and tissue regeneration after cell damage [21, 22]. Moreover, MSC-Exos can carry therapeutic drugs and other drugs to target cells, and nanoscale exosomes are more likely to reach damaged tissues after injection and have the advantages of low immunogenicity and tumorigenicity, thus attracting widespread attention [23, 24].

### Neurological regeneration

**Fig. 1:**
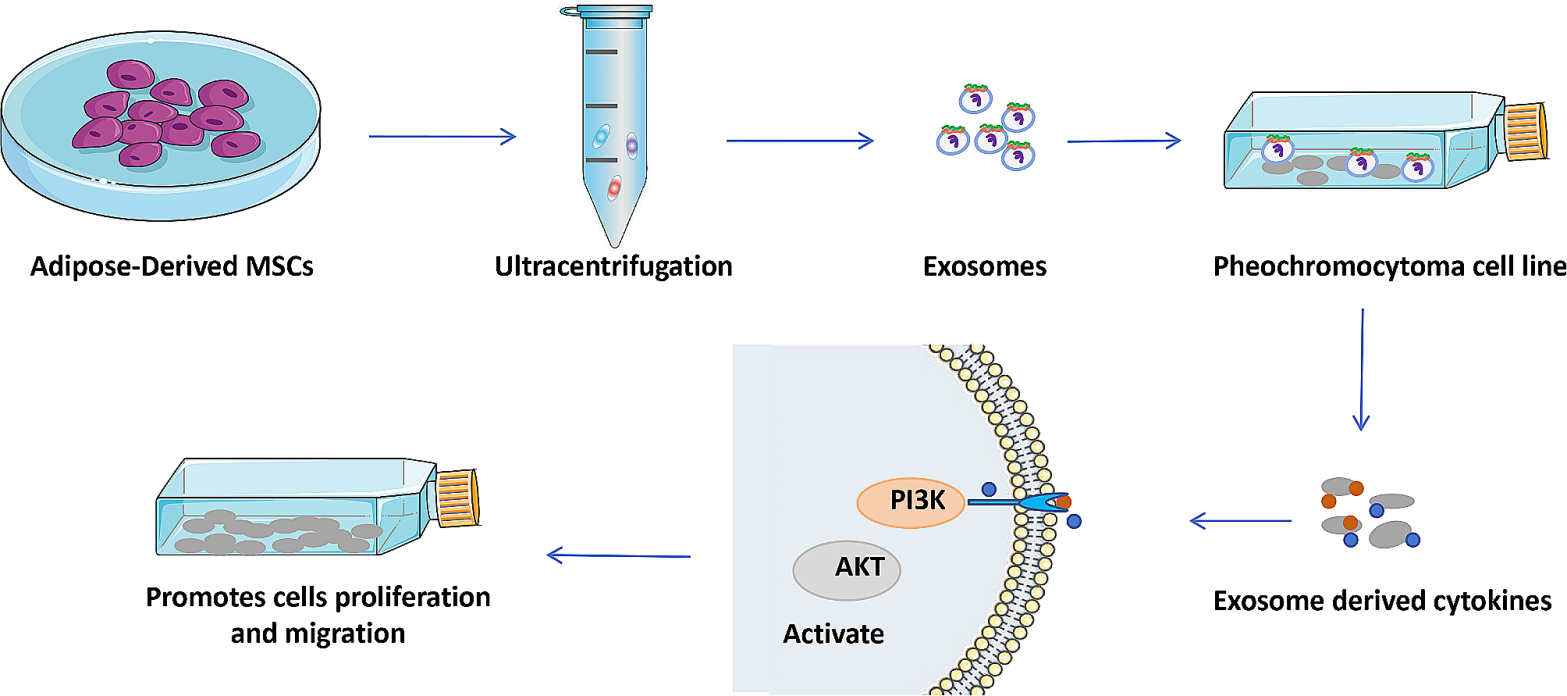
The role of mesenchymal stem cells derived exosomes in nervous system diseases. A model of glutamatergic neuronal cell injury established by exosomal treatment of PC12 cells with adipose mesenchymal stem cells. Exosomal release of cytokines (insulin growth factor, hepatocyte growth factor) enhances injured cell viability

MSC-Exos have shown therapeutic advantages in neurological disorders and neurodegenerative diseases. Xie et al. established a glutamatergic neuronal cell injury model using PC12 cells and treated the injured cells with Adipose-Derived MSC-Exo (ADSC-Exo). The results showed that the exosomes contained a large amount of cytokines that have some protective effect against brain injury, such as insulin growth factor and hepatocyte growth factor, and were able to improve the survival rate of the injured cells. The mechanism of action may be attributed to exosomes exerting a protective effect on neural injury through the PI3KAkt pathway [25] (Fig. 1). However, this study needs to be further investigated in terms of the association of PI3K/Akt signaling pathway, MSC-Exo and nerve injury repair.

Alzheimer’s disease (AD) is the most common cause of dementia in older adults, accounting for 60–80% of the total dementia population [26]. AD is a neurodegenerative disease characterized by β-amylopeptide deposition in the brain, and enkephalinase is the most important β-amylopeptide degrading enzyme in the brain. Adipose MSC-Exo are able to carry high levels of enkephalinase and reduce intracellular and extracellular β-amylopeptides when co-cultured with adult neuroblastoma cells that highly express β-amylopeptides, suggesting that adipose MSC-Exos may be effective in treating AD [27, 28]. However, all these results are in accordance with previous studies that describe the improvement of cognitive functional capacities in different animal models of brain damage. Some studies provide evidence that one of the mechanisms by which exosomes interact with neurogenic niches is by the transference of miRNAs to precursor neural precursor cells, triggering neural remodeling events, neurogenesis, angiogenesis and synaptogenesis. Neuroinflammation is an emerging central pathological process in AD [29]. Excessive accumulation of β amyloid in the brain triggers a neuroinflammatory process. MSC-Exos contribute to immunomodulation and neuroinflammation improvement in pathologically abnormal regions and significantly improve spatial learning ability and memory impairment in AD transgenic mice [30, 31]. In addition, MSC-Exos induces anti-inflammatory effects by inhibiting the release of activated microglia, reactive astrocytes and cytokines [32]. Despite promising results from previous trials, few ongoing and completed clinical studies have explored the potential role of MSC-Exos [33]. Researchers from Shanghai Jiao Tong University School of Medicine evaluated the safety and effectiveness of MSC-Exos in patients with mild-to-moderate dementia (NCT04388982). This clinical trial recruited nine patients. Patients were given three doses of low, medium, and high (5 µg, 10 µg, and 20 µg) MSC-Exos twice a week, respectively, for 12 weeks via nasal drip. In the primary stage of the trials, patients were assessed for liver or kidney function and treatment-related adverse events. Cognitive function, quality of life, and neuroimaging findings will be evaluated during the second stage of the trial. Therefore, the results of MSC-Exos in clinical trials for AD are promising.

Recently, researchers are focusing more on Parkinson’s disease (PD). The pathological features of PD are characterized by the loss of dopaminergic neurons in the substantia nigra [34]. Therefore, cell replacement therapy is a simple and potentially curative treatment. Recent research has shown that in a mouse model of PD, knockdown of the RNA-binding protein polypyrimidine tract-binding protein (PTB) in astrocytes can directly transform them into functional neurons and consequently effectively improve PD-related dyskinesia. Notably, antisense oligonucleotides that inhibit PTB have shown similar therapeutic effects [35]. Another study transplanted green fluorescent protein–labeled MSCs into a PD rat model and showed significant improvements in animal behavior; green fluorescent protein–labeled tyrosine hydroxylase-positive cells were found in the brain of the rat model. These results suggest that MSCs are likely to replace missing dopaminergic neurons and can potentially treat PD [36]. In a PD mouse model, MSC-derived exosomes were found to contribute to the recovery of PD by promoting ICAM1-related angiogenesis. These findings demonstrate that the exosome-ICAM1-SMAD3/P38MAPK axis can promote the angiogenesis of human brain microvascular endothelial cells, with possible therapeutic potential for PD [37]. The aim of MSC therapy is to replace degenerative dopaminergic neurons, thereby targeting the pathogenesis of PD. Autologous MSCs transplantation was not harmful to recipients. MSCs transplantation can regenerate and restore nerves in damaged tissues, thereby offering better outcomes in PD treatment [38]. Current experimental evidence suggests that MSCs derived from the bone marrow, adipose tissue, and cord blood significantly improve the symptoms of PD [39]. Several studies show that MSC-Exos are able to transfer miRNAs to neuronal cells, in which exosomes enriched in miR-133b can promote neurite outgrowth, which is of great benefit for PD, as it is one of the miRNAs that is normally downregulated in the disease [40].

Although MSC-Exos shows a role in neurological disorders and neurodegenerative diseases. However, as a new therapeutic tool, MSC-Exos still faces many challenges in clinical application, such as the need to continuously improve and standardize the method of exosome isolation and purification, to ensure the safety of exosome clinical application, and to enhance the comparability and reproducibility of clinical application. MSC-Exos may paracrine some harmful cytokines or chemokines, such as tumor necrosis factor alpha and interleukin 6 etc., which needs to be further explored in the exosomes as well as to eliminate the interference of unknown secretory factors. Therefore, in view of the diverse sources of MSC-Exos and the different outcomes they may cause, there are still many questions that need to be addressed in the future use of MSC-Exos in the treatment of neurodegenerative diseases.

### Cardiovascular regeneration

Paracrine effects of MSC-Exos have been reported in various diseases [41, 42]. Kang et al. investigated the effects of exosomes from rat bone marrow stem cells on cardiac function in a rat model of myocardial infarction [43]. They observed that exosomes protected cardiomyocytes from ischemic injury in vitro and in vivo by acting on the heart and blood vessels and promoting cardiac regeneration mediated by neovascularization and anti-vascular remodeling [44]. This study also showed that exosomes secreted by MSCs could reduce myocardial ischemia/reperfusion injury [44]. In addition, MSC-Exos overexpressing CXCR4 showed better efficiency in reducing left ventricular remodeling and promoting the recovery of cardiac function [43].

**Fig. 2:**
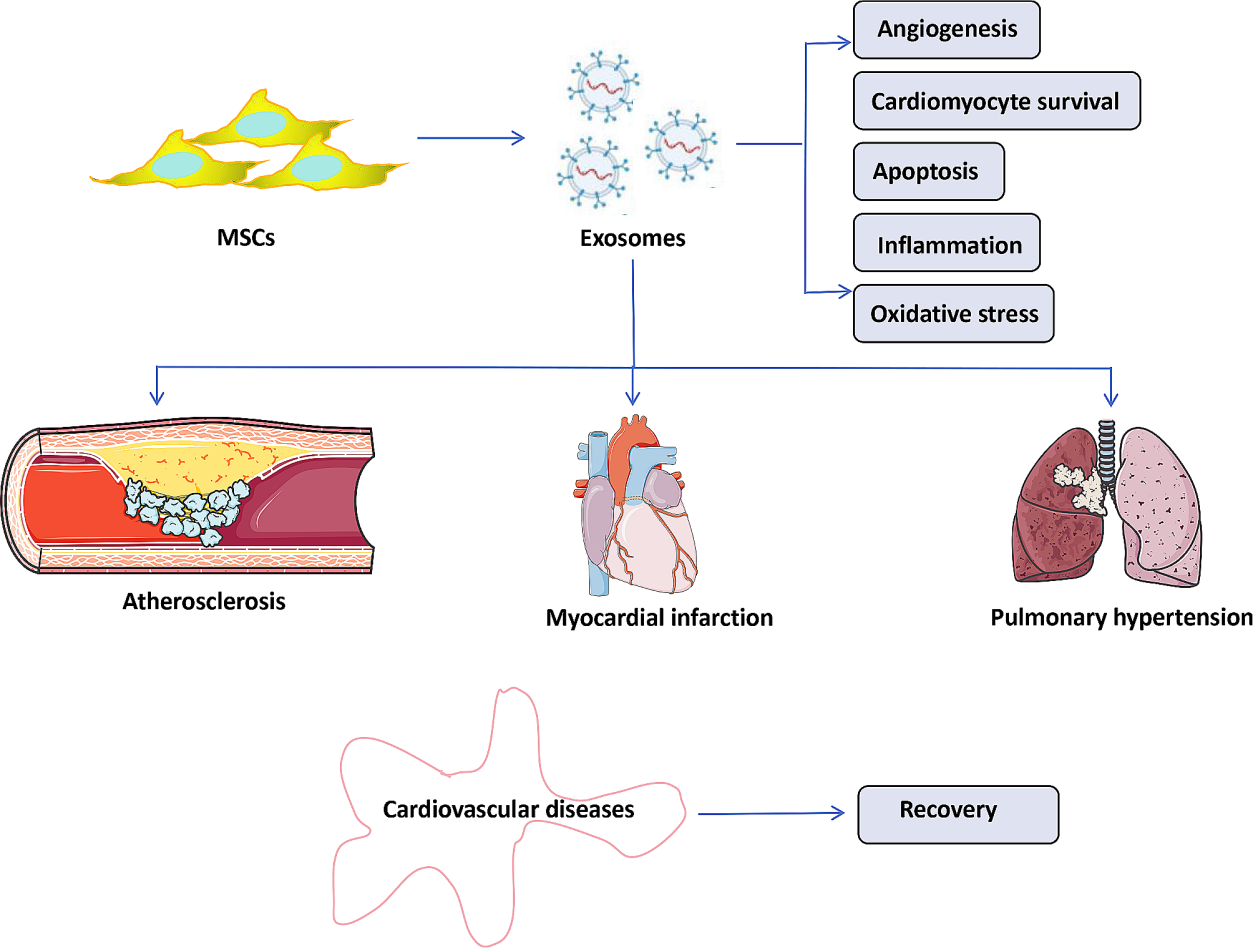
Mesenchymal stem cells derived exosomes exhibits potential therapy for cardiovascular diseases (CVDs). MSC-Exos protects the damaged tissues by inducing angiogenesis and survival of cells through different mechanisms including reducing oxidative stress, inflammation and apoptosis

Other studies have reported that MSC-Exos reduce oxidative stress, increase ATP and NADH production, control inflammatory activity, and activate the PI3K/Akt pathway, which in turn leads to protective effects on cardiomyocytes and the survival and preservation of left ventricular function after ischemia-reperfusion injury [45] (Fig. 2). Similarly, MSC-Exos reduced infarct size in a porcine model of ischemia-reperfusion injury [46]. In another in vivo study, an analysis of a mouse model of muscle injury showed that the injection of MSC-Exos accelerated muscle regeneration, enhanced angiogenesis, and reduced fibrosis [47]. Placental MSC-Exos have been shown to promote the migration and vascularization of placental microvascular endothelial cells [48]. In a similar study, the injection of MSC-derived exosomes into stroke rats reduced severe symptoms by stimulating angiogenesis, neuronal remodeling, and neurogenesis [49]. These studies demonstrate the potential role of MSC-Exos in vascular regeneration.

The above studies reveal that MSC-Exos play an essential role in MSCs-based therapy of cardiovascular diseases. Compared to traditional MSCs therapies, MSC-Exos therapies will decrease injury from MSCs transplantation surgery, avoid the risk of unexpected differentiation into other cell types such as osteoblasts, adipocytes and chondrocytes as well as vascular calcification, and reduce possibility of favoring tumor growth by MSCs. Accordingly, administration of in vitro purified MSC-Exos becomes more attractive than conventional stem cells transplantation. Effects of MSC-Exos on cardiovascular system should be largely attributed to functional cytokines, miRNAs and proteins in exosomes. Nevertheless, how to preserve biological activity of cytokines, miRNAs and proteins in exosomes and deliver them to target sites is a big challenge for us now. Following investigations are needed to solve the troubles of MSC-Exos therapy and make MSC-Exos become a promising entity for cardiovascular diseases.

### Kidney damage repair

Renal insufficiency rapidly develops because of acute kidney injury (AKI), with high mortality and morbidity rates [50]. In allogeneic kidney transplant patients, ischemia-reperfusion is a common cause of AKI and its complications, which are an unavoidable problem during transplantation [51–53]. Wan et al. found that MSC-Exos alleviate acute kidney injury by inhibiting pyroptosis in rats and NRK-52E cells [54]. Another study showed that treatment with human umbilical cord MSC-Exos decreased IRAK146 expression through upregulation of miR-1b levels, leading to inhibition of NF-κB activity and ultimately alleviating sepsis-associated AKI and improving survival in septic mice [55]. Human umbilical cord MSC-Exos may serve as novel therapeutic agents for reducing sepsis-associated AKI. In addition, Yu et al. demonstrated that human umbilical cord MSC-Exos could regulate necroptosis through miR-874-3p to attenuate renal tubular epithelial cell injury and enhance repair, providing new therapeutic modalities and possibilities for treating AKI and the process of AKI-to-CKD transformation to mitigate renal damage [56].

MSCs induce kidney transplantation tolerance by increasing T regulatory cells (Treg). MSC-Exos induce immune tolerance to mouse kidney transplantation by transporting long non-coding RNA differentiation, antagonizing non-protein-coding RNA [57]. Another study demonstrated the potential use of MSC-Exos in treating renal failure. Zhang et al. found that umbilical cord MSC-Exos alleviate kidney failure progression by modulating inflammatory responses and oxidative stress in an ischemia-reperfusion mouse model.

In conclusion, MSC-Exos therapy shows increasing potential for alleviating AKI. Research still faces significant challenges. There is no consensus regarding the reporting of studies using exosomes as it is an emerging therapeutic and there is a lack of global standardisation in isolation, characterization and validation protocols. Furthermore, clinical translation is in its infancy and the conclusions are limited to preclinical animal models. They are monocausal and simplistic when compared to the multifactorial aetiologies of AKI and comorbidities, such as increasing age and cardiovascular disease, from which patients suffer. For translation of exosomes therapy to clinical practice, the following manufacturing issues surrounding optimal dosing, mode of injection, schedule of administration, potency assays, minimising dose toxicity, uniformity between batches, identification of exosomes and safety must be standardized. Most studies used a single dose of exosomes, but this may be insufficient to achieve a sustained effect in humans. Multiple doses of exosomes showed greater efficacy than single dosing but repeated injections decrease feasibility. Future studies should engineer the surface and cargo of exosomes for superior specificity and develop optimal protocols for delivery and safe transition into clinical practice.

### Liver damage repair

Various factors, such as viral hepatitis, drugs, alcohol, and metabolic diseases, can cause liver damage, and MSC-Exos have been used as a treatment option for liver damage repair. After carbon tetrachloride-induced liver fibrosis in mice, direct intrahepatic injection of human umbilical cord mesenchymal stem cell exosomes was found to reduce hepatocyte transforming growth factor TGF-β1 expression, reverse hepatocyte epidermal mesenchymal transition by blocking Smad2 signaling pathway phosphorylation, and ultimately reduce collagen deposition and promote liver injury repair [58]. In another study, adipose-derived MSC-Exos were shown to suppress hepatocellular carcinoma growth in a rat model [59]. However, this animal study is only a short-term investigation that fails to show the long-term therapeutic impact of ADMSC-derived exosomes on hepatocellular carcinoma. In addition, several of the exosome-treated rats did not show a therapeutic response for reasons that remain to be elucidated. Further studies are needed to investigate the complex mechanisms and cellular molecular changes induced by MSC-Exos. CCl4 induced acute injury and promoted ferroptosis in the liver. Lin et al. found that MSCs protect against ferroptosis via the exosome-mediated stabilization of SLC7A11 during acute liver injury [60].

Accumulating evidence suggests that autophagy has many beneficial effects in preventing liver injury [61]. Hepatocytes can promptly remove damaged mitochondria through autophagy, thus blocking apoptosis induced by the mitochondrial pathway [62]. Zhao et al. established a hepatocyte injury and apoptosis model using LPS/D-GalN to investigate the protective effects and mechanisms of MSC-Exos on hepatocyte apoptosis [63]. The results showed that MSC-Exos treatment increased the expression of the autophagy marker proteins LC3-II and Beclin-1, thereby promoting the formation of autophagosomes and inhibiting hepatocyte apoptosis [63]. Lin et al. found that bone marrow derived MSC-Exos attenuate d-GaIN/LPS-induced hepatocyte apoptosis by activating autophagy in vitro [64]. Therefore, using drugs to increase autophagic activity may contribute to disease remission, thus providing a new therapeutic option for liver injury [65].

Unfortunately, there are few clinical investigations on MSC-Exos for the treatment of liver diseases. The potential of MSC-Exos exosomes for the treatment of liver disease is exciting, but the difficulties are challenging. Although MSC-Exos are relatively easy to obtain, MSCs themselves have a limited number of in vitro passages, which results in limited access to exosomes. Future applications of MSC-Exos are most likely in combination with other drugs or systems. Therefore, the therapeutic mechanisms of exosome intervention in disease onset must be further elucidated.

### Repair of articular cartilage

**Fig. 3:**
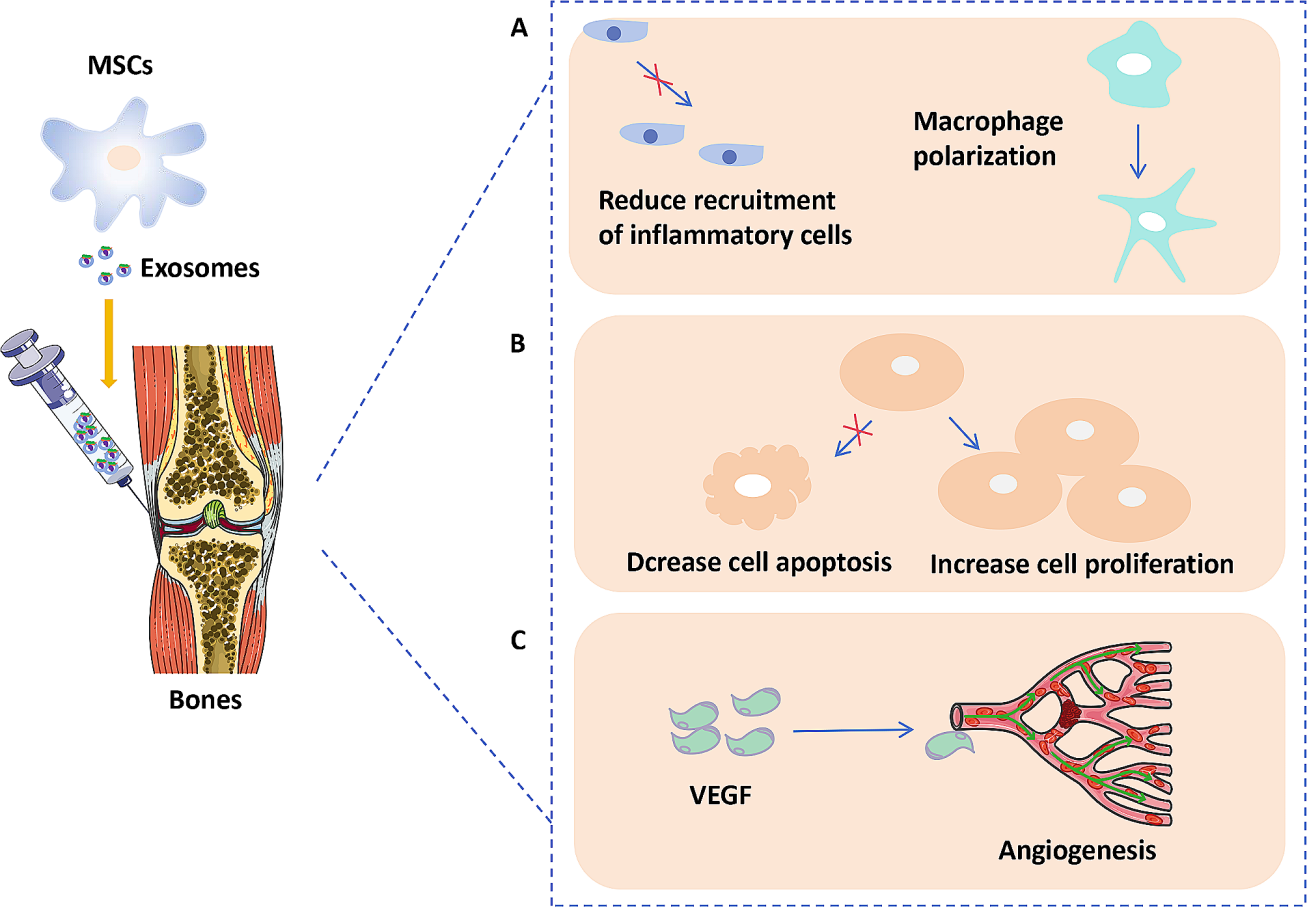
Mechanisms of mesenchymal stem cells derived exosomes RNA on bone injuries. A Exosomes could attenuate early inflammatory response via reducing inflammatory, polarizing macrophage from a pro-inflammatory M1 phenotype to a pro-regenerative M2 phenotype, and reducing inflammatory cytokines release. B Exosomes could enhance cell proliferation and reduce apoptosis. C Exosomes promote angiogenesis by activating the VEGF

Osteoarthritis (OA) is a systemic, chronic joint disease caused by the degeneration of articular cartilage and a major cause of disability in adults. It is characterized by the degeneration of articular cartilage, resulting in cartilage destruction, narrowing of the joint space, and subchondral sclerosis. Current treatment options for OA have limitations. Traditional medical treatments only control symptoms and delay injury but cannot reverse the progression of OA. MSCs therapy has been shown to be effective for cartilage repair in both animal studies and human clinical trials [66, 67]. The efficacy of the MSC-based therapeutic chondrogenic potential is increasingly attributed to paracrine and exocrine secretions. Liu et al. found that MSC-Exos promote the proliferation and inhibit the apoptosis of chondrocytes via the lncRNA-KLF3-AS1/miR-206/GIT1 axis in osteoarthritis [68]. Additionally, MSC-Exos-associated RNAs are involved in cartilage regeneration and degeneration in OA. One study found that MSC-Exos-mediated long non-coding RNA KLF3-AS1 repressed autophagy and apoptosis of chondrocytes in OA. Qiu et al. study found that MiR-129-5p shuttled by human synovial MSC-Exos relieved IL-1β induced osteoarthritis via targeting high mobility group protein-1 [69]. Thus, MSC-Exos are rich in different types of RNAs that are involved in cartilage regeneration through various pathways (Fig. 3).

Although the immunomodulatory and regenerative properties of MSC-Exos have demonstrated their potential in OA therapies, challenges such as sourcing, culture condition, preservation, and possible host conflicts after transplantation remain. MSC-Exos are considered a key factor for their small size, stability, biological activity, and targeting property in the treatment of OA. To further expand the therapeutic scope of MSC-Exos beyond their inherent functions, engineering approaches can significantly improve the efficacy of clinical OA indications. Although we have proposed many of the advantages of exosomes, the current research on exosome therapy for OA is limited to the preclinical animal phase. Two technical obstacles mainly limit the application of exosomes-on the one hand, current technology makes it difficult to obtain exosomes in a simplified and high yield; on the other hand, efforts are still needed to efficiently isolate functional exosomes. As work continues, the resolution of issues such as idealized sources of exosome, standardized access, delivery routes, dosing, and dosing frequency is key to bringing exosomes therapy for OA into the clinic setting.

### Oral tissue regeneration

Traditional treatments for pulp infection or necrosis (root canal therapy and apical barrier surgery) replace viable pulp with biological materials, whereas pulp vascular regeneration treatments only result in the formation of osteoid, bone-like tissue, and pulp-like and periodontal membrane-like connective tissue in the root canal, thus failing to achieve true pulp regeneration [70, 71]. Based on the three elements of tissue engineering, the selection of stem cells and scaffold materials and the induction of the local microenvironment (selection of cytokines) are urgent issues to be solved to truly achieve physiologically functional pulp regeneration, as exosomes are rich in bioactive factors that can replace single cytokines and play an important role in pulp regeneration [72]. Nakao et al. study found that exosomes from TNF-α-treated human gingiva-derived MSCs enhance M2 macrophage polarization and inhibit periodontal bone loss [73].

According to published scientific documents, MSC-Exos exert positive effects on teeth-protecting components [74]. In this regard, in vivo and in vitro studies have demonstrated that this type of exosome can potentiate periodontal tissue and bone regeneration and the proliferation and migration of periodontal ligament cells by triggering the AKT and ERK signaling pathways through the mediation of adenosine receptors [75]. Moreover, it has been implicated that human gingival MSC-Exos decrease inflammatory reactions in periodontal ligament stem cells triggered by lipopolysaccharides by inhibiting the NF-κB and Wnt5a signaling pathways [76]. Some reports have also stated that dental pulp MSC-Exos expedite the improvement of alveolar bone through mechanisms such as the transfer of miR-1246 to immune agents and the suppression of osteoclast formation [77, 78]. However, the yield of these exosomes is low owing to the isolation technique, culture system, and purification process [79]. It has been suggested that a three-dimensional (3D) culture system can provide more exosomes and elevate their therapeutic effects by transferring certain cargoes [80]. Zhang and colleagues utilized 3D-cultured dental pulp MSC-Exos and observed that these extracellular vesicles served as anti-inflammatory agents in an animal model of periodontitis by recovering the Th17 cell/Treg balance [81]. Evidence indicates that MSC-Exos can be effective therapeutic tools against periodontitis; however, further studies are required to confirm their efficacy against this disease.

In conclusion, the paragraphs emphasize the potential for therapeutic applications of MSC-Exos in addressing periodontal issues. MSC-Exos play a crucial role in mitigating the damaging effects of periodontitis by alleviating oxidative stress, inhibiting inflammation, and promoting tissue regeneration. Despite the fact that periodontal regeneration stimulating-influence of MSC-EVs on various animal designs have been assessed by preclinical investigations, clinical experiments for periodontitis mending are presently limited. To begin with, none of the standard techniques utilized for extraction and characterization investigation are generally approved. Moreover, the challenges associated with clinical administration including safety, forms of administration, dosage, rate of recurrence, drug movement within the body, and adverse reactions still need to be analyzed.

## Discussion

**Fig. 4:**
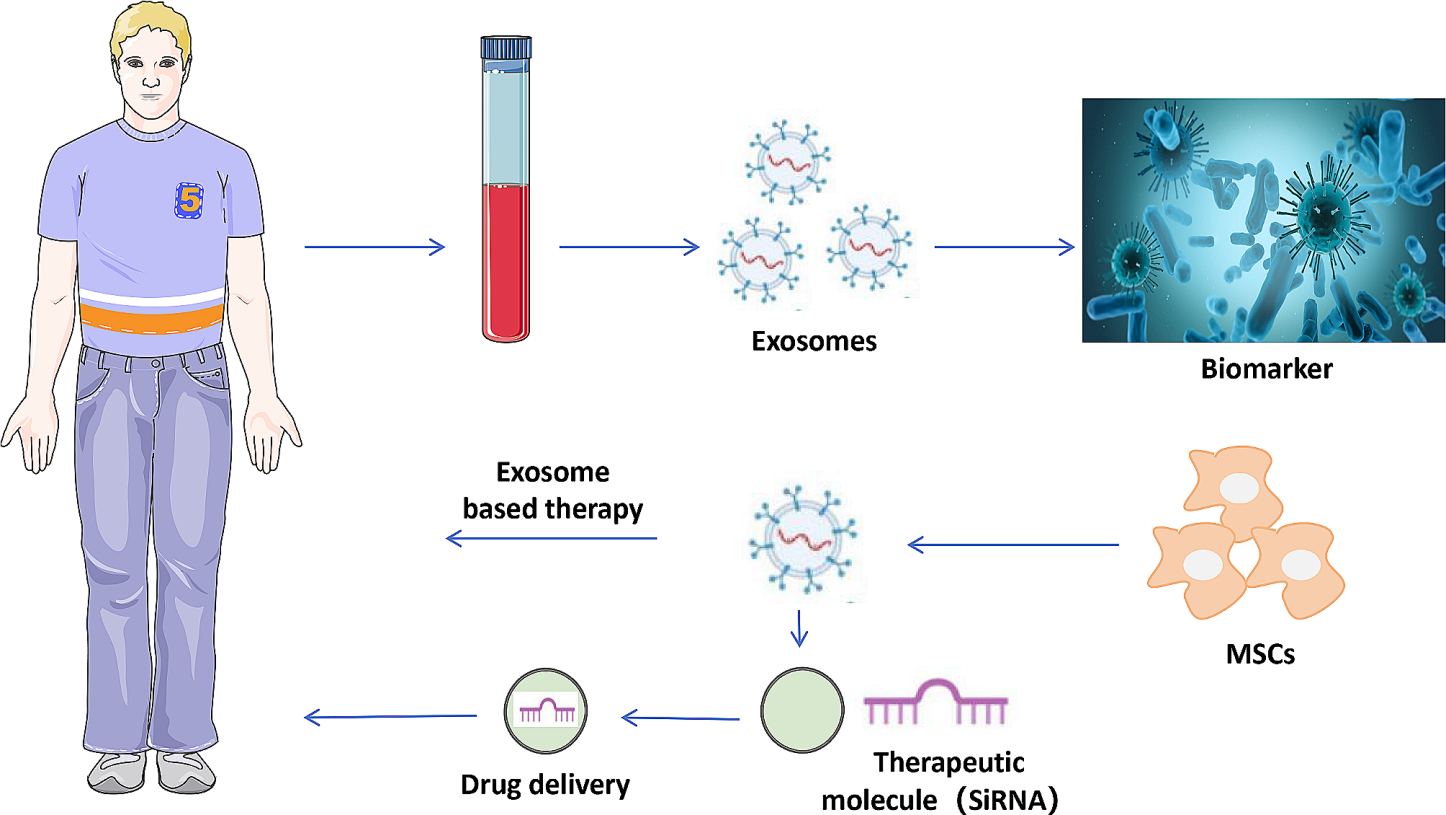
Potential therapeutic applications of MSC-Exos as biomarkers and drug delivery vehicle. MSC-EXOs have enormous potential as a biomarker and drug delivery vehicle for various diseases such as neurological, autoimmune and inflammatory, cancer, ischemic heart disease, lung injury, and liver fibrosis due to enhanced biocompatibility, excellent payload capability, and reduced immunogenicity compared to alternative polymeric-based carriers

This review highlights the therapeutic potential of MSC-Exos, which has received increasing attention as promising tools for cell-free therapies. We summarize in Table 1 some classical examples about the application of MSC-Exos in different tissue repairs. Overall, the results of various studies suggest the promising therapeutic potential of MSC-Exos, which can be used as drug delivery vectors and biomarkers in exosome-based therapies to treat various diseases [82] (Fig. 4). The important challenge for the application of MSC-Exos is to identify the molecules involved in the paracrine action of stem cells, which facilitates the opening of new therapeutic avenues using exosomes, including the production, processing, and manufacturing aspects, particularly the isolation, purification and characterization [83]. Although a variety of separation techniques and methods are available, such as ultracentrifugation [84], size exclusion [85], immunoaffinity capture [86], precipitation, and microfluidics [87, 88] the preparation of clinical-grade exosomes remains a key factor. Another important factor is that there is no uniform method for isolating exosomes and each method has its own advantages and disadvantages [89]. Therefore, the MSC-Exo separation method must be standardized. The most important obstacle is exosome production, which can be improved by combining currently available methods, such as 3D microcarrier-based suspension cultures facilitated by certain bioactive materials. To increase their production, exosomes need to be synthesized artificially; their composition is relatively controllable, and the therapeutic pathway is clear.

**Table 1:** Summarizes some classical examples about the application of MSC-Exos in different tissue repairs

MSCs source of Exos	Application/purpose	Method of Exos isolation	Effectiveness/results	References
Bone marrow mesenchymal stem cells	MSC-Exos can enhance neurogenesis and restore cognitive function lost by administration of Aβ1–42 aggregates	Ultracentrifugation	As a possible therapeutic strategy for the treatment of chronic degenerative neurologic diseases.	27
Human adipose mesenchymal stem cells	MSC-Exos were found to contribute to the recovery of PD by promoting ICAM1-related angiogenesis.	Ultracentrifugation	MSC-Exos can promote the angiogenesis of human brain microvascular endothelial cells, with possible therapeutic potential for Parkinson’s disease.	37
Rat bone marrow mesenchymal stem cells	Exosomes secreted from CXCR4 overexpressing mesenchymal stem cells promote cardioprotection via Akt signaling pathway following myocardial Infarction	Polymer coprecipitation method	Cardiac functions were improved by transplantation of exosome-treated MSC via increased angiogenesis in the infarcted heart.	42
Human umbilical cord mesenchymal stem cells	HucMSC-Exos can regulate necroptosis through miR-874-3p to attenuate renal tubular epithelial cell injury and enhance repair	Filtration + ultracentrifugation	This study provides new therapeutic modalities and ideas for the treatment of AKI and the process of AKI to CKD transformation to mitigate renal damage.	55
Adipose-derived mesenchymal stem cells(ADMSC)	Adipose-derived mesenchymal stem cell exosomes suppress hepatocellular carcinoma growth in a rat model	Filtration + ultracentrifugation	ADMSC-derived exosomes promoted NKT-cell antitumor responses in rats, thereby facilitating HCC suppression	58
Human mesenchymal stem cells	MSCs derived exosomal lncRNA KLF3-AS1 transplantation could facilitate cartilage repair by promoting chondrocyte proliferation and inhibiting apoptosis via miR-206/GIT1 axis	Polymer coprecipitation method + ultracentrifugation	Exosomes derived from mesenchymal stem cells have potential therapeutic effects on osteoarthritis	67
Human gingival tissue-derived MSCs	Exosomes from TNF-α-treated human gingiva-derived MSCs enhance M2 macrophage polarization and inhibit periodontal bone loss	Filtration + ultracentrifugation	TNF-*α*-preconditioned GMSC-derived exosomes may be a promising therapeutic tool against periodontitis	72

MSC-Exos have shown great potential because they appear to have the same beneficial characteristics of cell applications, such as the release of cytokines and growth factors. They are particularly used in tissue regeneration tests due to extensive evidence of their biogenesis, isolation, and characterization processes. Exosomes released from stem cells have been utilized for numerous therapeutic purposes and in the development of novel regenerative approaches. Stem cells exert protective effects against various diseases by regulating various pathological conditions. MSC-Exos offer a greater possibility for cell-free therapy than the parental MSCs because of their regenerative and anti-inflammatory effects. The unique properties of MSC-Exos, such as their immunomodulatory effects, allow for the treatment of different inflammatory and autoimmune diseases.

## Conclusions

Exosomes play a vital role in both physiological and pathological processes, as they are released by diverse cell types and can be found in nearly all body fluids [90]. Acting as messengers in cell-to-cell communication, exosomes play a vital role in normal physiological and pathological processes [91]. However, the most difficult aspects of exosome research are their isolation and acquisition. Addressing this challenge will significantly advance research on exosomes. Accordingly, a suitable method has been developed to capture exosomes directly from plasma, serum, or urine. This method uses a range of exosomal membrane proteins, making it possible to isolate exosomes with minimal sample preparation, thereby eliminating the need for vesicle separation. In addition, exosomes found in body fluids show significant heterogeneity, underscoring the importance of tracing their origin. Changes in the expression of specific components in these exosomes may provide insights into their origins. Future studies can further explore the possibility of classifying exosomes in body fluids based on their contents, similar to the classification of blood cells.

Overall, MSC-derived exosomes have significant potential for regenerating different tissues, including neural, cardiovascular, hepatic, renal, articular, and oral tissues. An in-depth study of the specific mechanisms of action of MSC-Exos in different tissues is essential for the health of the organism. Determining the biological properties of MSC-exos as a cell-free therapeutic tool is important for the regeneration of different tissues.

## Data Availability

No datasets were generated or analysed during the current study.

## Abbreviations

MSCs: Mesenchymal stem cells
MSC-Exos: Mesenchymal stem cell-derived exosomes
ADSC-Exos: Adipose-Derived MSC-Exos
AD: Alzheimer’s disease
PD: Parkinson’s disease
PTB: Polypyrimidine tract-binding protein
AKI: Acute kidney injury
OA: Osteoarthritis
3D: Three-dimensional
